# The dynamic relationship between the life-space mobility and family resilience of older adult patients with chronic heart failure

**DOI:** 10.3389/fpubh.2025.1677106

**Published:** 2025-10-27

**Authors:** Shuhong Yue, Yu Zhang, Xinyu Yang, Minyan Chen

**Affiliations:** ^1^Department of Nursing, Tongxiang First People’s Hospital, Tongxiang, Zhejiang, China; ^2^Department of Nursing, College of Medical Science, Huzhou University, Huzhou, Zhejiang, China

**Keywords:** chronic heart failure, family resilience, life-space mobility, older adults, longitudinal study

## Abstract

**Background:**

Chronic heart failure (CHF) is a significant global health issue with a rising prevalence among the older population. Family resilience (FR) and life space mobility (LSM) are crucial factors affecting the quality of home-based cardiac rehabilitation in older adult CHF patients. However, the dynamic interplay between these factors and their trajectories over time remain unclear. This study aims to explore the longitudinal relationship between FR and LSM in older adult CHF patients.

**Methods:**

This longitudinal study recruited 274 older adult CHF patients from January 2024 to June 2024, with a final sample size of 254 participants entering the study. Participants were assessed at three time points (1, 6, and 12 months post-discharge) using the Life-Space Assessment scale (LSA) and the Family Resilience Assessment Scale-Chinese Version (FRAS-C). Latent growth curve modeling and cross-lagged panel models were applied to analyze the dynamic relationship between FR and LSM.

**Results:**

Both family resilience and life-space mobility exhibited linear increases over time, with significant inter-individual variability in their initial levels and changes. A significant positive correlation was found between family resilience and life-space mobility across all time points (*p* < 0.05). The cross-lagged analysis revealed a bidirectional predictive relationship between family resilience and life-space mobility, with family resilience showing a stronger predictive effect on life-space mobility (*p* < 0.05). Sensitivity analysis confirmed the robustness of these findings, demonstrating the stability of the results.

**Conclusion:**

This study emphasizes the importance of family resilience in enhancing life-space mobility and improving the quality of life for older adults with CHF. Clinical interventions should prioritize strengthening family support systems to optimize patient outcomes and foster greater mobility.

## 1 Introduction

Chronic heart failure (CHF) represents a growing global public health challenge, affecting more than 64 million individuals worldwide, with its prevalence increasing steadily, largely due to population aging (1). CHF is defined by a progressive decline in cardiac pumping function, resulting in systemic fluid retention, diminished exercise capacity, and substantial impairment in quality of life (2). In severe cases, the one-year mortality rate exceeds 50%, and five-year survival rates are comparable to those observed in certain cancers (3). In the United States, CHF contributes to approximately one in nine deaths and accounts for over $30 billion in annual direct medical costs (4), highlighting its significance as a critical public health issue.

Among older adults, CHF progression is often complicated by comorbidities such as hypertension, diabetes, and cognitive decline, which further impair functional capacity. These individuals frequently experience marked reductions in mobility and independence, limiting their ability to perform essential daily activities (5). Life-space mobility (LSM), a dynamic indicator of an individual’s movement within both domestic and community settings, is significantly restricted in older adults with CHF (6). Unlike controlled clinical assessments, LSM reflects real-world physical functioning, encompassing both indoor mobility and participation in community-based activities. Physical limitations, particularly reduced walking endurance, lead to progressive restrictions in LSM, diminishing social engagement and increasing reliance on caregivers (7). These limitations are also associated with adverse mental health outcomes, including depression, anxiety, and social isolation, which further complicate disease management (8).

Family caregivers play a crucial role in CHF management by supporting medication adherence, physical care, and emotional well-being. Caring for older adult CHF patients is a long-term commitment, with many caregivers spending more than 5 h daily on caregiving tasks (9). This sustained responsibility contributes to high levels of psychological distress, including depression and burnout, among caregivers (10). Combined with physical strain and insufficient social support, caregiving stress can weaken FR—the capacity of a family system to adapt to adversity and maintain functionality under chronic stress (11). Research indicates that higher FR is linked to improved coping strategies for patients and greater caregiver capacity to manage ongoing care demands, which is essential in CHF management (12, 13).

Although numerous studies have examined LSM and FR separately in the context of CHF, their interplay remains poorly understood. Most existing research adopts a cross-sectional design, focusing on physical impairments and their effects on FR without exploring the longitudinal bidirectional relationship between LSM and FR. For instance, Peng et al. (14) demonstrated that social support and effective coping strategies enhance caregiver resilience, which is vital for CHF patient care. Similarly, Hashimoto et al. (15) reported that lower life-space assessment scale (LSA) scores predict worse prognoses in older adult cardiovascular patients, yet the connection between LSM and FR has not been investigated. These findings underscore the need to examine the dynamic interaction between LSM and FR in CHF patients and to understand how these factors jointly influence disease outcomes.

This study aims to explore the dynamic relationship between LSM and FR in older adults with chronic heart failure. Specifically, the research objectives are: (1) to characterize the developmental trajectories of LSM and FR over time; (2) to investigate the bidirectional influences between LSM and FR throughout the disease course; and (3) to assess how FR predicts improvements in LSM and how optimized living environments affect FR. By addressing these knowledge gaps, this research seeks to inform integrated strategies that combine environmental and familial interventions to improve long-term care outcomes for older adult CHF patients.

## 2 Materials and methods

### 2.1 Data sources and subjects

**Figure 1:**
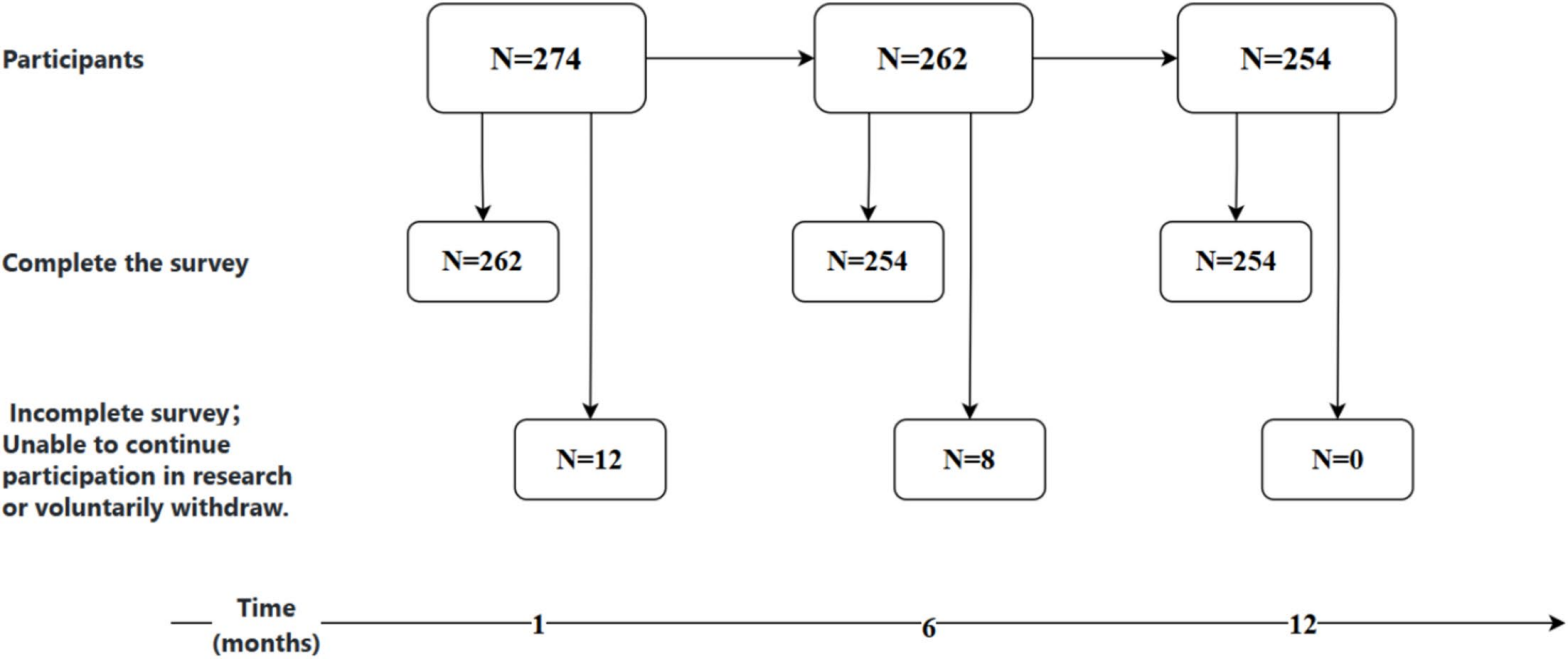
Study participant inclusion flowchart.

The study employed a convenience sampling method, selecting older adult patients with chronic heart failure (CHF) admitted to the cardiology department of Tongxiang City First People’s Hospital from January 2024 to June 2024 as the subjects of the research, with a one-year follow-up period. The inclusion criteria were as follows: (1)aged 60 years or older; meeting the diagnostic criteria for CHF as outlined in the “Chinese Guidelines for the Diagnosis and Treatment of Heart Failure 2024” (16) and having stable conditions;(2) no mental disorders, with normal memory and communication abilities; (3) and providing informed consent and voluntarily participating in the study. The exclusion criteria included: (1) patients with concurrent malignant tumors; (2) those readmitted to the hospital unplanned within 1 month after discharge; (3) patients lost to follow-up after discharge; (4) and those with other diseases affecting mobility (such as lower limb fractures). The study conducted surveys on eligible CHF patients at four time points: 1 month (T1), 6 months (T2), and 12 months (T3) after discharge. Utilizing the sample size estimation method for repeated measures designs, with an assumed average correlation coefficient of 0.5, an effect size (*f*) of 0.14, a significance level (*α*) of 0.05, and a power (*β*) of 0.2, the calculated sample size required was 86 participants. Accounting for an estimated 20% attrition rate, the minimum required sample size was adjusted to 103 participants. In the initial phase of the study, a total of 274 participants were recruited. By the time of T1, 12 individuals had either not completed the survey or were lost to follow-up; by T2 (6 months), an additional 8 individuals were lost; and by T3 (12 months), no further dropouts occurred. Ultimately, 254 participants completed all three follow-ups, resulting in an overall attrition rate of 7.3% (Figure 1).

### 2.2 Procedure

In this research endeavor, staff members, rigorously trained to a uniform standard, initiated contact with participants via telephone to gage their interest in engaging in either hospital-based consultations or in-home evaluations. In instances where direct interaction was impractical, videoconferencing was utilized to facilitate the survey. Upon completion of recruitment, investigators employed convenience sampling to conduct in-person interviews within community health settings, meticulously distributing and collecting questionnaires while offering continuous support and elucidation. To avert potential biases, a series of strategic measures were implemented during the data collection phase: firstly, data collectors were subjected to a standardized training regimen, with a particular focus on employing neutral language; secondly, participants encountering difficulties with reading or comprehension were offered a detailed, question-by-question reading and explanation service; thirdly, a tranquil and secluded environment was ensured for the completion of questionnaires or the conduct of interviews; and lastly, the confidentiality of data was underscored, emphasizing its exclusive use for scientific research, thus diminishing the influence of social desirability bias in responses.

### 2.3 Ethics considerations

Participants were briefed on the study’s aims, methodology, potential risks, benefits, and confidentiality measures, and acknowledged that their involvement was optional. Ethical clearance for this research was obtained from the Medical Ethics Committee of Tongxiang First People’s Hospital (Approval number: 2025–015-01).

### 2.4 Measurements

#### 2.4.1 Life-space assessment scale

The Life-Space Assessment scale (LSA), translated by JI and others, is used to assess the activity range of Chinese older adult (17). The scale assesses an individual’s spatial mobility over the past month, covering five different areas from other rooms in the home to outside the town. The scale integrates the activity distance, frequency, and independence of the older adult, with a score ranging from 0 to 120, the higher the score, the broader the activity space. The Cronbach’s *α* coefficient of the scale is 0.76.

#### 2.4.2 Family resilience assessment scale-Chinese version

The Family Resilience Assessment Scale-Chinese Version (FRAS-C) employed in this study is a psychometric instrument translated and adapted by Li et al. (18) for use in Chinese-speaking populations. The scale encompasses three dimensions: family communication and problem-solving, utilization of social resources, and maintenance of a positive attitude, comprising a total of 32 items. It utilizes a 4-point Likert scale, ranging from “strongly disagree” to “strongly agree,” with scores assigned from 1 to 4, respectively. The total score ranges from 32 to 128, with higher scores indicating a higher level of family resilience. The scale demonstrated good internal consistency, as evidenced by a Cronbach’s *α* coefficient of 0.960.

#### 2.4.3 Covariates

In this study, potential confounding effects were accounted for by including the following covariates: age groups (60–70 years, 70–80 years, 70–74 years, ≥80 years), gender (male, female), educational attainment (illiterate, primary school, junior high school, high school or vocational school, university or above), housing type (ground-level or single-story houses with stairs, two or more stories with elevators, two or more stories with stairs), multiple chronic conditions (yes, no), marital status (married, other), per capita monthly household income (<3,000 CNY, 3000–5,000 CNY, >5,000 CNY), living alone (yes, no), residential area (urban, rural), cognitive dysfunction (yes, no), and lower limb dysfunction (yes, no). “Multiple chronic conditions” refers to the coexistence of two or more chronic diseases in a patient. Cognitive function was assessed using the Mini-Mental State Examination (MMSE). Patients were considered to have cognitive dysfunction if they scored ≤13 for illiterate individuals, ≤19 for those with 1–6 years of education, and ≤24 for individuals with 7 or more years of education (19). Lower limb function was evaluated using the Fugl-Meyer Motor Function Assessment Scale, with scores below 34 indicating lower limb dysfunction and scores of 34 or above indicating normal lower limb function (20). Heart function is assessed using the NYHA classification proposed by the New York Heart Association, which categorizes it into four levels: I to IV (21).

### 2.5 Statistical methods

Statistical analyses were conducted using SPSS 27.0 and Mplus 8.7 software. Quantitative data were categorized into normally distributed and non-normally distributed variables, with the former described by $\bar{x}\pm s$ and the latter by *M*(*P_25_, P_75_*). Categorical data were presented as frequencies and percentages. To assess differences in general data between genders, we employed independent samples t-tests, Mann–Whitney U tests, and chi-square tests. To examine changes in cognitive function and muscle mass among older adult at different time points, we utilized repeated measures analysis of variance (ANOVA). Correlations between variables were assessed using Pearson or Spearman correlation coefficients. Latent growth curve modeling (LGCM) was applied to fit the trajectories of changes in living space and family resilience; cross-lagged panel models (CLPM) were constructed to investigate the temporal causal relationships between living space and family resilience. The level of significance was set at *α* = 0.05.

## 3 Results

### 3.1 General information of older adult patients with chronic heart failure

Our study enrolled 254 participants who completed three follow-ups over the course of 1 year. The cohort included 108 males (42.52%) and 146 females (57.48%), with the majority aged between 70 and 80 years (43.7%). Most participants had completed primary education (42.91%), resided in bungalows or first-floor apartments without elevators (43.31%), were married (79.53%), and had an average monthly income between 3,000 and 5,000 yuan (70.08%). A smaller percentage lived alone (11.42%) or were classified as urban older adult (31.1%). Cognitive impairment was identified in 24.8% of the older adult, and lower limb dysfunction in 12.99%. Notably, the majority of older adult had heart function classified at NYHA Class III (66.53%). For further details, refer to Table 1.

**Table 1:** General Information (*N* = 254).

Characteristics		*N* (%)	Characteristics		*N* (%)
Age	60 ~ 70 years old	85 (33.46)	Marital status	Married	202 (79.53)
	70 ~ 80 years old	111 (43.7)		Others (single, divorced, widowed)	52 (20.47)
	≥80 years old	58 (22.83)	Living alone	Yes	29 (11.42)
Gender	Male	108 (42.52)		No	225 (88.58)
	Female	146 (57.48)	Place of residence	Urban	79 (31.10)
Education level	Illiterate	71 (27.95)		Rural	175 (68.90)
	Primary school	109 (42.91)	Cognitive dysfunction	Yes	63 (24.80)
	Junior high school	23 (9.06)		No	191 (75.20)
	High school or technical secondary school	31 (12.20)	Lower limb dysfunction	Yes	33 (12.99)
	College or above	20 (7.87)		No	221 (87.01)
Housing type	Bungalows or first-floor apartments without elevators	110 (43.31)	NYHA classification	I	0 (0.00)
	Second-floor or above apartments with elevators	79 (31.10)		II	62 (24.41)
	Second-floor or above apartments without elevators	65 (25.59)		III	169 (66.53)
Multiple chronic conditions	Yes	158 (62.21)		IV	23 (9.06)
	No	96 (37.79)			
Per capita income (CNY)	<3,000	52 (20.47)			
	3,000 ~ 5,000	178 (70.08)			
	>5,000	24 (9.45)			

### 3.2 Correlation analysis between living space and family resilience in older adult patients with chronic heart failure

**Figure 2:**
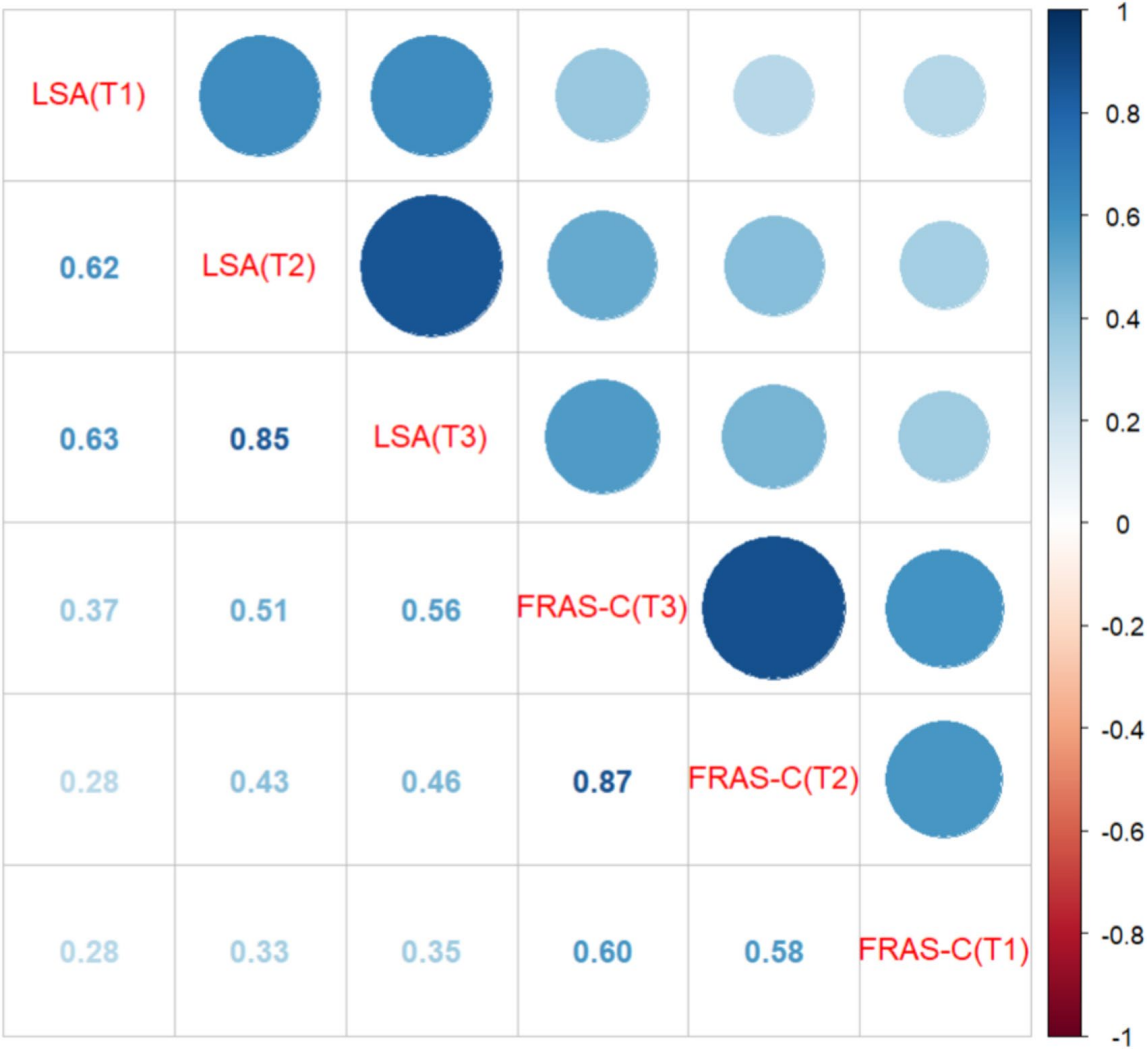
Correlation analysis (*N* = 254). The numbers in the figure represent correlation coefficients, with all *p*-values being less than 0.05.

Table 2 presents the correlation between living space and family resilience among older adult patients with CHF, revealing statistically significant score differences. For more details, see Table 2. The correlation analysis shows a significant positive correlation between the two variables at the same time point (0.284 ≤ *r* ≤ 0.564, *p* < 0.05) and across different time points (0.284 ≤ *r* ≤ 0.871, *p* < 0.05). For further details, refer to Figure 2.

**Table 2:** Analysis of differences in patients’ living space and family resilience at different time points (*N* = 254).

Items	T1	T2	T3	*p*
Living space	44.83 ± 21.31	51.7 ± 18.21^①^	61.24 ± 20.26^①②^	<0.05
Family resilience	86.16 ± 20.22	89.23 ± 18.43^①^	90.28 ± 21.45^①②^	<0.05

Compared with T1,^①^ *p* < 0.05; Compared with T2, ^②^*p* < 0.05.

### 3.3 Developmental trajectories of living space and family resilience among older adult patients with chronic heart failure

In our study, given that the number of survey time points was less than 4, we deemed linear models to be a robust choice (22, 23). Consequently, we developed two unconditional linear latent growth models to examine the developmental trajectories of living space levels and family resilience among older adult patients with chronic heart failure.

In the case of the developmental trajectory of family resilience among older adult patients with CHF, the model fit indices presented a *χ^2^/df* ratio of 2.957, a Comparative Fit Index (*CFI*) of 0.996, a Tucker-Lewis Index (*TLI*) of 0.987, a Root Mean Square Error of Approximation (*RMSEA*) of 0.090, and a Standardized Root Mean Square Residual (*SRMR*) of 0.021, suggesting an adequate fit of the model. The mean initial level of family resilience was 86.425, and it showed a linear increase across the three measurement points, with a slope of 1.455. The variance in the intercept was significantly different (*p* < 0.05), indicating significant inter-individual differences in the initial levels of family resilience, while the variance in the slope was not significantly different (*p* = 0.099), implying minimal inter-individual variability in the rate of change of family resilience. The correlation between the intercept and slope factors was 0.545, which was not statistically significant (*p* = 0.126), indicating no significant association between the initial level and the rate of change of family resilience.

**Figure 3:**
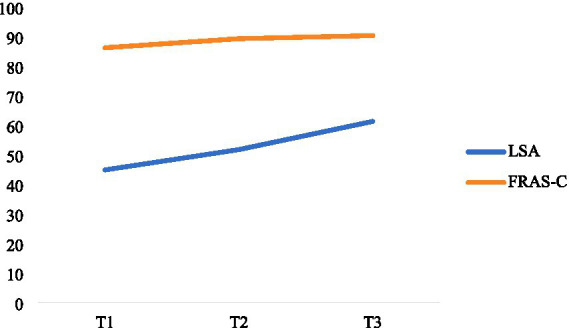
Latent growth curve model diagram of FRAS-C and LSA.

For the trajectory of living space levels in this patient population, the model fit indices revealed a *χ*^2^/*df* ratio of 1.030, a *CFI* of 1, a *TLI* of 1, a *RMSEA* of 0.011, and a *SRMR* of 0.013. These metrics indicate an excellent fit of the model to the data. The mean initial level of living space was 42.305, and it demonstrated a linear increase across the three measurement points, with a slope of 8.770. Notably, both the intercept and slope variances were significantly different (*p* < 0.05), highlighting substantial inter-individual variability in the initial levels and rates of change of living space among older adult patients with CHF. Moreover, the correlation between the intercept and slope factors was 0.355, which was not statistically significant (*p* = 0.318), suggesting no significant association between the initial level and the rate of change of living space. The specific trajectories of change are shown in Figure 3.

### 3.4 CLPM analysis of living space and family resilience in older adults with chronic heart failure

**Figure 4:**
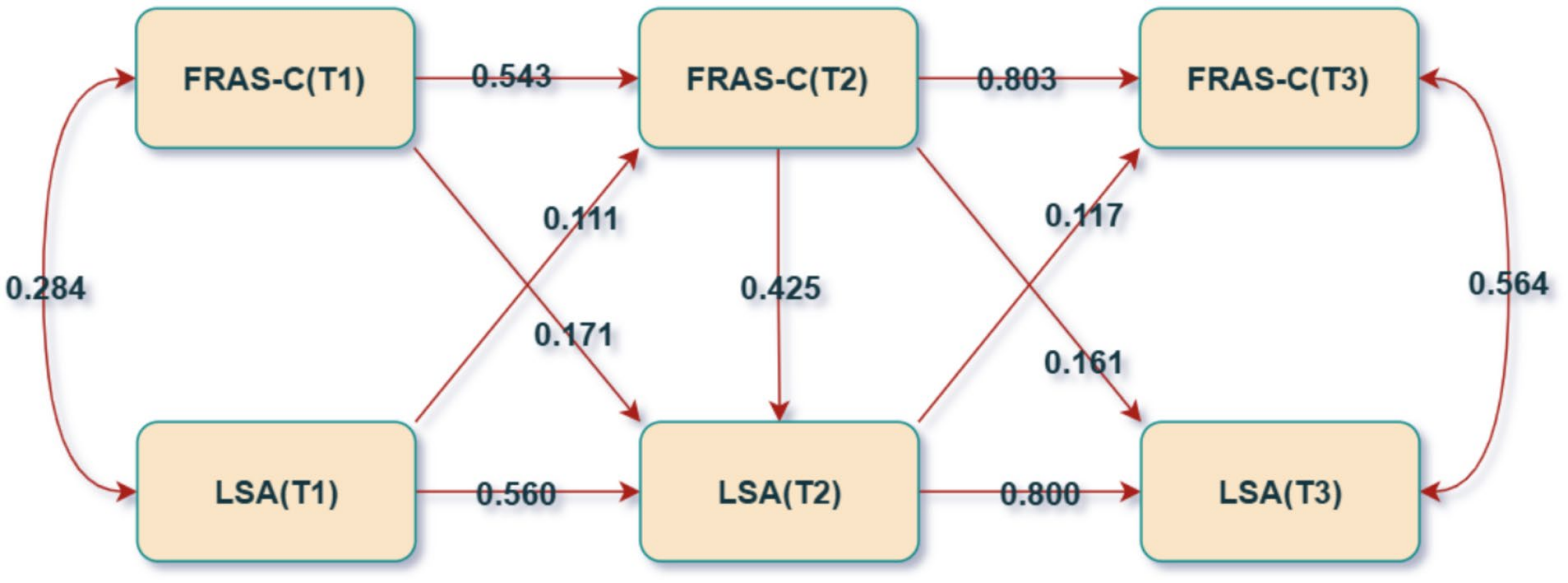
Cross-lag model (*N* = 254). Double-headed arrows indicate correlations, while single-headed arrows represent regressions; each present solid line signifies a p-values less than 0.05.

In the study examining the causal relationship between living space and family resilience among older adult patients with CHF, we constructed four path models: M1, M2, M3, and M4. M1 served as the baseline model, including only the autoregressive effects of the independent variable, living space, and the dependent variable, family resilience. M2 expanded upon M1 by adding predictive paths from living space to family resilience at the subsequent time point. M3 built upon M1 by incorporating predictive paths from family resilience to living space at the subsequent time point. M4, the full model, encompassed autoregressive paths, as well as bidirectional predictive paths from living space to family resilience and vice versa. Each model controlled for factors such as age, gender, education level, housing type, marital status, per capita income, living alone, place of residence, cognitive dysfunction, and lower limb dysfunction. The results indicated that M4 outperformed the others in terms of fit indices, leading to its selection as the final model for further analysis. For more in-depth information, see Table 3 and Figure 4. The findings from the M4 model revealed that the path from living space to family resilience was statistically significant (*p* < 0.05) at both the T1 to T2 and T2 to T3 time intervals, with a positive path coefficient *β*, suggesting that improvements in living space can predict enhancements in family resilience. Conversely, the path from family resilience to living space was also statistically significant (*p* < 0.05) at both time intervals, with a positive path coefficient *β*, indicating that increased family resilience can predict improvements in living space. Moreover, the path coefficient for the family resilience to living space was greater than that for the living space to family resilience, indicating a stronger predictive effect of family resilience on living space. Collectively, these results suggest a reciprocal causal relationship between family resilience and living space (Table 4).

**Table 3:** Model fit.

Model	*χ* ^2^	*df*	χ^2^/*df*	*CFI*	*TLI*	*SRMR*	*RMSEA*
M1	88.461	9	9.829	0.914	0.866	0.184	0.192
M2	59.429	7	8.490	0.943	0.886	0.134	0.177
M3	65.21	7	9.316	0.937	0.874	0.127	0.187
M4	21.227	4	7.786	0.981	0.935	0.024	0.006

**Table 4:** Path Coefficients (*N* = 254).

The path	T1 → T2	T2 → T3
LSA autoregressive	0.560**	0.800**
FRAS-C autoregressive	0.543**	0.803**
LSA → FR	0.111**	0.117**
FRAS-C → LSA	0.171**	0.161**

*p < 0.05, **p < 0.01.

### 3.5 Sensitivity analysis

Following the exclusion of patients with lower limb dysfunction, a sensitivity analysis was conducted in this study. The cross-lagged model results were re-examined, revealing a good model fit with *χ^2^/df* = 5.2, *CFI* = 0.981, *TLI* = 0.934, *SRMR* = 0.024, and *RMSEA* = 0.005. The specific path coefficients are presented in Table 5. The findings from the sensitivity analysis were largely consistent with those of the primary analysis, thereby attesting to the robustness of the study’s outcomes.

**Table 5:** Sensitivity analysis path coefficients (*N* = 254).

The path	T1 → T2	T2 → T3
LSA autoregressive	0.571**	0.812**
FRAS-C autoregressive	0.546**	0.812**
LSA → FRAS-C	0.131**	0.141**
FRAS-C → LSA	0.168**	0.158**

*p < 0.05; **p < 0.01.

## 4 Discussion

This longitudinal study provides important insights into the bidirectional relationship between FR and LSM among older adults with CHF. The findings reveal that both FR and LSM exhibit a linear upward trend within the first 12 months following hospital discharge, with FR predicting improvements in LSM and LSM reciprocally influencing FR. Notably, the effect of FR on LSM was stronger, indicating that a strong family support system plays a crucial role in enhancing mobility and activity levels among older CHF patients.

The gradual increase in FR observed in this study (*S* = 1.455, *p* < 0.05) is consistent with the resilience model, which suggests that individuals can reduce the negative impact of stress through adaptive coping strategies and external support systems. In the context of CHF, families provide essential emotional and practical assistance, thereby enhancing patients’ ability to manage physical limitations. Prior research has underscored the role of FR in improving quality of life and psychological well-being among individuals with chronic illnesses (24, 25). Interestingly, no significant correlation was found between baseline FR levels and their rate of development (*p* = 0.099), indicating that even patients with initially low FR can substantially improve through targeted interventions. This highlights the importance of implementing proactive support programs for families to better manage daily caregiving challenges (26).

The study also identified a progressive improvement in LSA scores among older CHF patients after hospital discharge. However, LSA scores remained below those of community-dwelling older adults, whose average score was 63.54 ± 20.83 (27). This discrepancy may be attributed to prolonged disease duration, reduced cardiac function, and long-term activity restrictions experienced by CHF patients. Age-related physiological decline further compounds these limitations, restricting activities primarily to the home environment (28–30). Moreover, no significant association was observed between initial LSM levels and the rate of change (*p* = 0.318), suggesting that even individuals with limited baseline LSM can benefit from timely interventions. Therefore, healthcare providers should enhance post-discharge guidance, including recommendations for daily physical activity, symptom monitoring, and dietary management, to facilitate better adaptation to the home setting.

This study establishes a significant bidirectional predictive relationship between FR and LSM in older adult patients with CHF. Although the effect size is not large, the statistical significance and consistent bidirectional pattern indicate that this relationship is systemic rather than coincidental. This reveals that family function intervention could be a potential leverage point for improving patients’ mobility capabilities. Even if the effect size is modest, from a public health perspective, the small but sustained improvements in patients’ mobility achieved through family system interventions could also have cumulative and significant value. FR contributes to maintaining functional independence by providing consistent assistance with daily tasks and offering psychological protection. This reduces patients’ anxiety about mobility and enhances motivation to engage in outdoor activities (11, 25). While expanding LSM may positively influence family functioning through increased social engagement (31), its effectiveness is constrained by the high dependency of older CHF patients on familial support (32, 33). Unlike LSM, which is susceptible to acute symptom fluctuations, FR serves as a durable, long-term protective factor against activity restrictions caused by disease progression. The underlying mechanism is that high family resilience not only provides instrumental support that promotes going out (6), but also fosters a family atmosphere that encourages activity by alleviating the burden on caregivers (34). Furthermore, it enhances the patient’s sense of self-efficacy through positive communication, thereby reducing their fear of engaging in activities (35).

Therefore, clinical interventions should focus on strengthening FR through family-centered disease education, community-based care networks, and structured family support plans to optimize patient outcomes. Specific interventions, such as family counseling, support groups, and tailored family training programs, could be developed to strengthen family resilience and enhance patient mobility. These interventions can provide families with the tools and support needed to manage the caregiving challenges associated with CHF. In patients with diabetes and stroke (36, 37), studies have similarly found that family resilience is a significant predictor of patients’ physical function recovery, activity participation, and quality of life. This underscores that the findings of this study resonate with research in these areas, collectively indicating that “family functioning” is a key modifiable target that spans various chronic diseases and affects patient outcomes.

This study is the first to apply a cross-lagged model to examine the dynamic interplay between LSM and FR in CHF patients, offering valuable implications for clinical practice and future research. After interviewing CHF caregivers, Durante recommended a comprehensive intervention strategy to strengthen FR (38). Chiang stabilized family dynamics through home care interventions involving remote monitoring and phone consultations (39). Gary enhanced FR further by implementing family-centered aerobic and resistance training. Essentially, healthcare providers should leverage family support to alleviate the physical and emotional stress on CHF patients and caregivers, ensuring the stability of the family system (10).

Several limitations must be considered when interpreting the results of this study. First, as a single-center study, the findings may not be generalizable to other populations with different demographic or cultural characteristics. Future studies should aim to include multi-center cohorts across diverse regions to validate these results. Second, the sample was limited to older adults with CHF, which may not fully capture the variability in resilience and life-space mobility in other chronic disease populations. Future research should consider subgroup analyses, such as by severity of CHF, socioeconomic status, or urban/rural residence. These factors may influence the changes in FR and LSM, and examining these differences could provide valuable insights into developing more tailored interventions. Additionally, the model did not account for all potential influencing factors, including major comorbidities, cardiovascular history, guideline-directed medical therapy, and device therapy, which could introduce residual confounding. This study relied on self-reported measurements; while the FRAS-C and LSA are validated in Chinese-speaking older adults, their applicability in different subcultures warrants further investigation. Future studies could benefit from incorporating objective measurement tools to improve the accuracy and generalizability of the results.

## 5 Conclusion

This study utilizes latent variable growth modeling and cross-lagged modeling to explore the developmental trajectories and dynamic interactions between family resilience (FR) and life-space mobility (LSM) in older adults patients with chronic heart failure (CHF). The results indicate a linear increase in both FR and LSM, with a bidirectional predictive relationship between the two. Notably, the influence of FR on LSM is particularly pronounced, suggesting that family resilience plays a critical role in improving the life-space mobility of older adults CHF patients. These findings underscore the importance of focusing on enhancing family resilience in clinical practice, as it may contribute to expanding life-space mobility and improving the overall quality of life for older adults patients with CHF.

## Data Availability

The raw data supporting the conclusions of this article will be made available by the authors, without undue reservation.
